# Age-Associated Changes in Gut Microbiota and Dietary Components Related with the Immune System in Adulthood and Old Age: A Cross-Sectional Study

**DOI:** 10.3390/nu11081765

**Published:** 2019-07-31

**Authors:** Nuria Salazar, Silvia Arboleya, Tania Fernández-Navarro, Clara G. de los Reyes-Gavilán, Sonia Gonzalez, Miguel Gueimonde

**Affiliations:** 1 Instituto de Productos Lácteos de Asturias (IPLA-CSIC), Villaviciosa, 33300 Asturias, Spain; 2 Diet, Microbiota and Health Group, Instituto de Investigación Sanitaria del Principado de Asturias (ISPA), 33011 Oviedo, Spain; 3 Department of Functional Biology, University of Oviedo, 33006 Oviedo, Spain

**Keywords:** intestinal microbiota, adults, elderly, ageing, nutrient intake, diet

## Abstract

The fecal microbiota plays an important role in human health, and alterations in the microbiota–host interaction seem to be involved in the ageing process. Therefore, it is of interest to develop strategies for promoting a balanced microbiota in old age in order to prevent the physiological and immune decline associated with age. However, the specific microbiota changes in the transition from adulthood to senescence are not yet well understood. Here we assessed the levels of some intestinal microorganisms and short chain fatty acids (SCFAs) across different age-groups. In total, 153 adults from four age groups (<50, 50–65, 66–80, and >80 years-old) were recruited; the levels of different bacterial groups in fecal samples were determined by quantitative polymerase chain reaction (qPCR), and those of SCFA by gas chromatography. Dietary information was collected by using a Food Frequency Questionnaire. The presence of the *Bifidobacterium*, *Faecalibacterium*, *Bacteroides* group, and *Clostridium* cluster XIVa decreased with age up to 66–80 years of age, with differences reaching statistical significance for the latter group. Interestingly, the levels of some of these microorganisms recovered in the very old age group (>80 years), with these older individuals presenting significantly higher counts of *Akkermansia* and *Lactobacillus* group than adults and the younger elderly. In addition, ageing was associated with a progressively and statistically significant reduction in the fecal concentrations of SCFAs. Dietary intakes also showed some statistically significant differences among the groups for some macro- and micronutrients. Moreover, associations of some microorganisms with age and macronutrients were also evidenced. Considering the role that fecal microbiota alterations may have in terms of impairing homeostasis and resilience, our results underline the importance of understanding the ageing and immunosenescence processes by including the microbiota perspective.

## 1. Introduction

The gastrointestinal tract is home to a myriad of microbes, constituting the so called “intestinal microbiota” which is critical for health maintenance [1]. The adults’ microbiota is dominated by the phyla Bacteroidetes and Firmicutes, whilst Actinobacteria, Proteobacteria, and Verrucomicrobia are represented in lower percentages [2]. In addition to bacterial and eukaryotic cells, the main representative of Archaea in the adult gut is the methanogenic species *Methanobrevibacter smithii* [3]. Some alterations in the composition of this microbiota are linked to an increased risk of different diseases during both infancy and adulthood [1]. During adult life, in the absence of external disturbances, the fecal microbiota is relatively stable, but an altered composition has been repeatedly reported in elderly subjects [4]. This has attracted the interest of researchers with respect to the potential role of the microbiota in the ageing process and the possibilities for its modulation, given the growing proportion of elderly subjects in modern societies [5]. Extending the period of good health in seniors, with the concomitant reduction of the ageing-associated health-care costs, is a pending task and one of our main current societal challenges. Unfortunately, our understanding of the intestinal microbiota (IM) changes along ageing is still limited; therefore, improving our knowledge on the transition from adulthood to old age is essential for developing IM-targeted strategies for promoting healthy ageing.

Different studies have reported an alteration in the fecal microbiota composition in the elderly [6,7,8,9,10]. In this way, reduced levels of *Bacteroides* and *Clostridium* cluster XIVa have been repeatedly observed [6,10,11,12]. Similarly, important intestinal microorganisms, such as *Faecalibacterium prausnitzii* and *Bifidobacterium*, have also been found at lower levels in elderly subjects whereas others, such as *Akkermansia*, have been reported to be increased [10,13,14]. These age-related changes in the composition of the IM lead to a reduction in the intestinal levels of short chain fatty acids (SCFAs) [10]. Moreover, the immunity has also been repeatedly reported to be affected by ageing with a pro-inflammatory state and with increased levels of pro-inflammatory cytokines often present in elderly subjects [10,15]. These two aspects of ageing, microbiota and immune changes, are very likely related. On one hand, this inflammation may produce reactive oxygen species and, thus, a more oxidative intestinal environment with a concomitant decrease in strict anaerobes and an increase of facultative microorganisms. However, on the other hand, the intestinal microorganisms may also reduce the intestinal environment limiting these reactive species and therefore reducing inflammation. 

Interestingly, studies on extremely old subjects (centenarians) do not fully support the above mentioned age-associated alterations in the microbiota and immunity. This points out at a microbiota remodeling in very old individuals that may be different to that occurring in younger elder subjects [7,16]. These data suggest that extremely old people may have a specific IM profile, distinct from that of elders not living so long, allowing the extremely old to postpone major age-related conditions [17]. However, it should be noted that this delay in the appearance of health problems and the altered microbiota pattern are very likely influenced by the dietary and health habits in these long-living individuals [18]. In this regard, as previously stated, several functions are affected in elderly people, including the immune system and the microbiota, but the dietary habits are also different from those of younger adults [10]. Although the nutritional requirements of active elderly subjects may be similar to those of adults, their ability to absorb nutrients may be hampered, the appetite may be reduced, and chewing difficulties may be present, often resulting in a poor nutritional status. 

Therefore, the potential changes in the fecal microbiota composition and, more specifically, in the microbiota–diet interaction associated with ageing constitute a research area of great interest. Most of the studies on this area have compared elderly subjects with control groups composed of young adults, but there are limited data focusing specifically on the transition from adulthood to old age. Understanding this transition will allow the development of intervention strategies targeted at mature adults or young elderly, aiming at promoting healthy ageing. In this context, here we aimed at characterizing the levels of the main intestinal microorganisms, SCFAs, and nutritional habits in four different age groups ranging from adults (<50 years of age) to elderly of advanced age (>80 years of age).

## 2. Materials and Methods 

### 2.1. Volunteers and Samples

The study sample included 153 adults who were categorized into four age groups: <50 (*n* = 49), 50–65 (*n* = 58), 66–80 (*n* = 19), and >80 (*n* = 27) years. All volunteers were recruited in the central area of the Asturias Region (northern Spain). Exclusion criteria were previous diagnosis of gastrointestinal cancer, autoimmune diseases, digestive diseases or morbid obesity, and consumption of probiotics, prebiotics, or antibiotics during the previous month (Supplementary Table S1). Fecal samples were collected, immediately frozen and transported to the laboratory for microbiota analyses. At the laboratory, fecal samples were melted, homogenized (1:10 *w*/*v*) in phosphate-buffered saline (PBS), and the DNA and fecal supernatants were obtained as previously described [19]. Purified DNA and fecal supernatants were stored at −20 °C until analyses. The study was approved by the Regional Ethical Committee of Asturias Public Health Service and the Ethical Committee of CSIC; informed written consent was obtained from all volunteers participating in the study. 

### 2.2. Quantification of Fecal Microbiota Groups by Quantitative Polymerase Chain Reaction (q PCR)

Previously described primers and conditions [20] were used for quantification of the different bacterial populations analyzed (*Akkermansia*, *Bacteroides* group, *Bifidobacterium*, *Clostridium* cluster XIVa, *Lactobacillus* group, and *Faecalibacterium*). qPCR reactions were performed in a 7500 Fast Real-Time PCR System (Applied Biosystems, Foster City, CA, USA) using SYBR Green chemistry (Applied Biosystems). Bacterial levels were determined by including standard curves made with pure cultures of appropriate strains. Samples were analyzed in duplicate in at least two independent PCR runs. Bacterial levels were calculated as the log of the number of cells per gram of feces.

### 2.3. Analysis of SCFAs in Feces by Gas Chromatography-Mass Spectrometry/Flame Injection Detector (MS/FID)

For SCFA determination filtered (0.2-µm pore-size filters) fecal supernatants were submitted to gas chromatography in a chromatographic system, composed of a 6890NGC module with an FID and a MS 5973N detector (Agilent Technologies. Inc, santa Clara, CA, USA), as previously described [21].

### 2.4. Nutritional Assessment

Dietary intake was assessed by a personal interview for filling a validated semi-quantitative annual Food Frequency Questionnaire (FFQ) as described elsewhere [22]. In brief, the volunteers were interviewed by a trained dietician in an item-by-item manner whether they usually ate each food and, if so, how much they usually ate (three different serving sizes of each cooked food were presented in pictures) and about their cooking habits. Food intake was analyzed for energy, macronutrients, and fiber by using the nutrient Food Composition Tables developed by the Centre for Higher Studies in Nutrition and Dietetics (CESNID) [23] and for the consumption of polyphenols by using the Phenol-explorer Database [24]. The weight and height were recorded following standardized methods and the data were used to calculate the body mass index (BMI) through the formula weight (kg)/height (m^2^).

### 2.5. Statistical Analyses

Analysis of varianza (ANOVA), followed by post-hoc (Bonferroni test) analyses, was conducted for comparing the different microbiological variables analyzed among the four age groups. Differences, according to age groups, were estimated by multivariate analysis of variance including gender, BMI, and energy as covariates. When significant effects were observed the Bonferroni test was used for inter-groups comparison. Pearson correlation test was used for assessing associations between age/macronutrients and microbiota as continuous variables. Differences in the proportions of categorical variables were assessed by chi-square test. Analyses were performed with R software (version 3.3.3, The R Foundation, Vienna, Austria) and the conventional *p*-level of 0.05 was used in the interpretation of results.

## 3. Results

**Figure 1:**
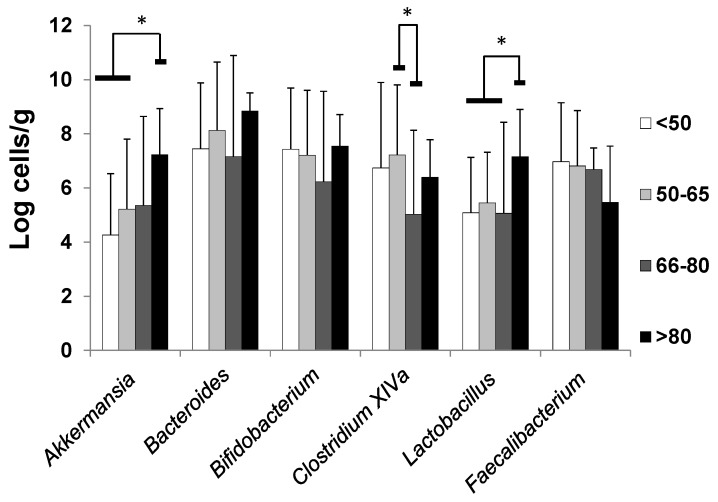
Bacterial levels (log cells/g, estimated marginal mean ± SD) of the microbial groups analyzed in the different age groups. Results were adjusted by energy intake, gender and BMI. * Denotes statistically significant differences (*p* < 0.05) among the indicated age groups.

The general characteristics of the individuals according to age-groups included in this study are shown in Table 1. Regarding the fecal microbiota composition (Figure 1), the *Bacteroides* group was the dominant among the bacteria analyzed in all age groups, with levels ranging between 6.9 ± 3.7 log cells/g feces (mean ± SD) for subjects of 66–80 years and 8.8 ± 0.7 log cells/g feces (mean ± SD) in the oldest individuals (over 80 years of age). When the levels of these microorganisms were compared among the different age groups (Figure 1), the oldest group (>80 years of age) was found to harbor significantly higher levels of *Akkermansia* and *Lactobacillus*-group than the younger age groups. In addition, significantly lower levels of *Clostridium* cluster XIVa were found in the group aged 66–80 years as compared with the younger (50–65 years) adult group. No other statistically significant differences where observed, likely due to the large inter-individual variability. Nevertheless, it is worth mentioning that the levels of the *Bifidobacterium*, *Faecalibacterium,* and *Bacteroides* groups were lower with increasing age of the different population groups up to the group aged 66–80 years, but surprisingly, this was not the case in the very old age group (>80) for which the levels of all these microorganisms, with the only exception of *Faecalibacterium*, were higher as compared with elderly subjects of 66–80 years of age. 

As expected, acetate was the major SCFA in the feces of all volunteers, followed by propionate and butyrate. The fecal concentration of SCFAs decreased along the age groups (Table 2). In contrast with that evidenced for the quantification of the different microbial groups, the concentration of the main SCFAs decreased with the increase of age, with the very old age group presenting the lowest fecal concentration of these compounds. This led to significantly (*p* < 0.05) lower levels of total SCFAs and individual SCFAs analyzed in the oldest group (>80 years of age) as compared with the young adult group (<50 years of age) (Table 2). 

Regarding nutritional habits and dietary intake, subjects between 66–80 years of age had a significantly (*p* < 0.05) lower energy intake than those in the 50–65 year old group (1624 ± 435 vs. 2033 ± 512 kcal/day, respectively), these two groups not differing significantly from those younger than 50 years (1922 ± 575 kcal/day) or over 80 years of age (1728 ± 400 Kcal/day). Due to this difference in energy intake this variable was used as a covariate, together with gender and BMI, for further analysis. As could be expected, differences in the intake of the major food groups and nutrients, some of them with influence on the immune system, were also observed among the age groups. Considering food categories, the intake of sugar and sugary products, vegetables, potatoes, fruit, fish and seafood and non-alcoholic drinks varied among the groups of age (Table 3). Consequently, fiber intake and lipid profile also showed differences among age groups. The intake of saturated and polyunsaturated fatty acids was higher in the groups of elderly over 80 years of age, whereas no differences between the extreme groups were observed for fiber intake. No other differences in macronutrients intake were observed among the analyzed age groups. However, regarding the micronutrients and compounds evaluated for their relationship with the immune system, differences among age groups were observed for the intake of carotenes, folic acid, vitamin A, vitamin C, vitamin D, vitamin B_6_, and polyphenols, the intakes being, in general, lower in the oldest group. 

**Figure 2:**
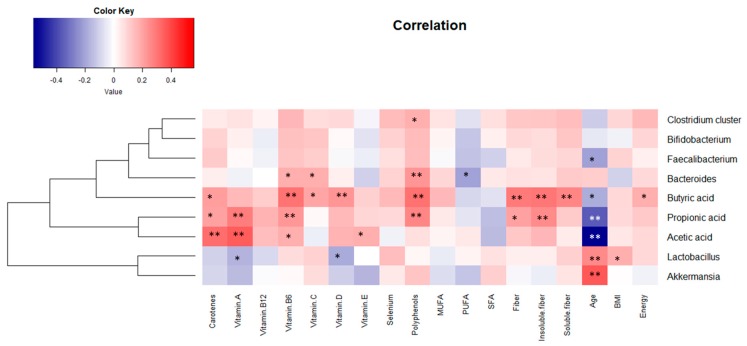
Pearson correlation between age and dietary compounds with the intestinal microbial groups and short chain fatty acids (SCFAs). The intensity of the colors represents the degree of association between the variables measured and asterisks indicate significant associations. * *p* < 0.05; ** *p* < 0.01. SFAs, saturated fatty acids. MUFAs, monounsaturated fatty acids. PUFAs, polyunsaturated fatty acids.

The correlation analyses performed between microbial groups and age showed a significant (*p* < 0.05) and positive correlation of age with levels of *Akkermansia* and lactobacilli, and a negative correlation (*p* < 0.05) with those of *Faecalibacterium* (Figure 2). As expected from microbial data, significant correlations (*p* < 0.05) between age and the fecal levels of SCFAs were also observed, in all cases being of negative sign and significant for the three major SCFAs. With regard to macronutrient–microbiota correlations, the intake of polyunsaturated fatty acids (PUFAs) correlated (*p* < 0.05) negatively with the levels of *Bacteroides* group, whereas consumption of vitamins B_6_ and C showed a statistically significant (*p* < 0.05) positive correlation with the levels of this microbial group (Figure 2). Similarly, polyphenols intake was positively correlated with the levels of *Bacteroides* and *Clostridium* cluster XIVa. On the contrary intake of vitamins A and D showed a statistically significant (*p* < 0.05) negative correlation with *Lactobacillus* group. Given that the origin of SCFAs in the colon is mainly the microbial fermentation of complex carbohydrates, it is not surprising that the levels of SCFAs, especially of butyrate and propionate, showed a positive association with the intake of fiber (either soluble or insoluble) and polyphenols. In this regard, it must be pointed out that polyphenols and fiber share their main food sources and, therefore, it is frequent to find common associations to both groups of compounds [21].

## 4. Discussion

In general, the intestinal bacterial levels determined in this study are within the range of those previously reported by us and other authors for similar human groups of age [6,10,25]. Although our study does not provide a complete microbiota characterization, such as that obtained by using Next-Generation Techniques (NGS) techniques, it does provide truly quantitative data instead of relative proportions, shedding additional light on the age group comparisons conducted. Moreover, our results confirm the ageing-related decline in the levels of some microorganisms such as the *Bacteroides* group, *Bifidobacterium, Faecalibacterium,* or *Clostridium* cluster XIVa previously reported [6,10,11,12,26,27]. This holds true for the group of elderly between 66 and 80 years of age, which matched the age range of human populations participating in most of the studies cited formerly, but strikingly, it is not true for the oldest subjects (over 80 years of age). In fact, the oldest group in our study showed higher levels of all the microorganisms analyzed, with the exception of *Faecalibacterium*, than the elders between 66 and 80 years, with these differences reaching statistical significance for *Akkermansia* and *Lactobacillus*. This suggests, in accordance with other authors [7,16,28], that very long-living individuals present a microbiota profile distinct from that of elderly subjects not living so long. The data reported by other authors, together with our observations, point out at the potential association of the presence of high levels of some of these microorganisms, such as *Akkermansia*, with a longer survival [29].

Moreover, our data on the comparison of the elderly (group aged 66–80 years) with those over 80 shed some light on the apparent lack of agreement between studies in elderly subjects. Apparently contradictory results have been previously reported for some of the fecal microbiota groups analyzed, such as *Akkermansia* [6,30] or lactobacilli [10,11,31], among others. To this regard the different methodologies used among the studies may have an effect. Nevertheless, on the view of our results, the age of the elderly groups participating in the different studies should be also considered as an important factor of variation, since differences among them are apparent. Moreover, although the different studies published, like this one, have defined inclusion criteria it is not always possible to ascertain the lack of effects due to treatments or conditions that were present in the life of the individual before running in the study.

Contrary to that expected from the microbiota data, the levels of SCFAs showed a continued age-associated reduction, with the oldest age group (subjects over 80 years of age) displaying SCFA levels that were less than half of those found in younger adults (<50 years old). This reduction in the amount of SCFAs found by us in elderly subjects confirms previous observations [10,31]. Given the important role played by the intestinal SCFAs [32] this reduction on the levels of these compounds during ageing may have profound consequences in terms of gut barrier maintenance and host physiological homeostasis. Interestingly, this differential trend of microbial levels and concentration of microbial metabolites in subjects over 80 years of age, suggests that, despite bacterial levels remaining high, there is a drop in the metabolic activity of the microbiota in these very old subjects. However, it is also important to underline that the SCFA fecal levels may also be influenced by other factors such as the the carbon sources available from diet or the absorption/excretion of these metabolites, which may also be affected in subjects of advanced age.

Our results also suggest potential associations between nutrient intake and the gut microbiota. In accordance with that previously reported [33] fiber associated positively with SCFA levels, whereas the polyphenols, compounds known to promote an appropriate immune status [34], correlated with the levels of some microorganisms. Some differences in fiber intake, including soluble fiber, are apparent among age groups; however, these are within a narrow range, which together with the lack of correlation observed between fiber and microbial groups in the correlation analyses suggest that the variation in fiber intake does not have a major role in the observed differences. In this regard, it is important to take into consideration that some dietary polyphenols cannot just be metabolized by the microbiota but they can affect the microbiota composition [35], even modulating some bacterial groups with a non-negligible role in the host immune system. Therefore, another aspect that is of great relevance in this sort of study is the potential impact of nutritional differences in the observed effects. Our data, in agreement with previous reports [9,10], show significant differences in dietary habits and, therefore, nutritional status among the analyzed age groups. In this sense it is also important to aknowledge that, although we do not think this has a significant effect in our results, a certain recall bias in relation to age, affecting the accuracy of the dietary information collected, may be present. Taking into account that distinct population groups, such as the elderly, may have different nutritional needs, the potential influence of such differences may not be neglected. Despite malnutrition in the elderly being linked with frailty and an increased risk of disease, this situation does not seem to be extrapolable to our sample, where the intake of macronutrients is similar across age groups and is sufficient to satisfy the nutritional requirements of each life stage [36,37]. However, some micronutrients and bioactive compounds are crucial for the maintenance of immunocompetence [38]. At this point, it is noteworthy that the group of subjects aged >80 years-old presented mineral intakes comparable to the younger groups (including iron, for which intake was above the recommended 8 mg/day for this age group), and even higher daily intakes of vitamins B_12_ and E as compared the rest of the groups. Nevertheless, the detection, in the group of people over 80 years of age, of a lower intake of some micronutrients with well documented roles in promoting immune function in the elderly, such as carotenoids, folic acid, and vitamins A and D, may be considered in future studies aiming to prevent/minimize immunosenescense by means of specific nutritional supplementation [4,39]. Also, polyphenols could play an additional key role in the complex bidirectional relationship between diet and immunity by means of microbiota modulation throughout life. Therefore, nutritional assessment and monitoring represent a key aspect when studying the age-associated changes in gut microbiota composition, and also in other aspects such as immune senescence. Such multifactorial approach promises to allow the development of nutritional strategies to positively modulate the fecal microbiota and immune system and to reduce nutritional deficiencies in the elderly. It is important, however, to underline that cross-sectional studies, like this one, present some limitations for the monitoring the shifts in the fecal microbiota along the lifespan. They may carry over important cohort effects and, while useful for understanding some phenomena and drawing hypotheses, validation from longitudinal studies is often needed.

## 5. Conclusions

Our results provide evidence on ageing-related changes in the composition and metabolic activity of the fecal microbiota as potentially beneficial for healthy ageing, signaling that specific microorganisms are linked to a long life span. Dietary changes are also present, which makes it difficult to discriminate microbiota changes attributed to ageing from those that could be a consequence of dietary or physiological modifications. In this context, it is important to understand the interaction between gut microbiota, diet, and ageing, since this knowledge promises to expand our possibilities for the development of diets and foods in order to promote healthy ageing.

## Figures and Tables

**Table 1:** General characteristics of subjects according to age groups.

Variables	Age Group (Years)			
	<50 (*n* = 49)	50–65 (*n* = 58)	66–80 (*n* = 19)	>80 (*n* = 27)
Female (%)	65.3	69.0	78.9	77.8
Age	37.6 ± 6.4	58.1 ± 4.0	72.8 ± 5.2	85.7 ± 4.4
BMI (kg/m^2^)	27.89 ± 5.21 ^a^	25.64 ± 3.19 ^b^	29.69 ± 4.31 ^a^	28.33 ± 3.98 ^a,b^

Results are presented as mean ± SD. Differences in categorical variables were examined using chi-squared analysis and are presented as a percentage (%). BMI: body mass index. The values of the same row not sharing the same superscript are significantly different, *p* < 0.05.

**Table 2:** Concentration (mM; estimated marginal mean ± SD) of acetate, propionate, butyrate and total short chain fatty acids (SCFAs) (acetate + propionate + butyrate) in fecal samples from volunteers of the different age groups.

SCFA	Age Group (Years)			
	<50	50–65	66–80	>80
Acetate	49.4 ± 18.3 ^a^	32.9 ± 14.7 ^b^	31.6 ± 17.7 ^bc^	18.8 ± 11.4 ^c^
Propionate	16.0 ± 7.8 ^a^	12.6 ± 5.9 ^ab^	12.1 ± 8.1 ^ab^	8.1 ± 6.8 ^b^
Butyrate	10.9 ± 5.3 ^a^	10.4 ± 8.1 ^ab^	12.1 ± 8.3 ^ab^	6.3 ± 6.4 ^b^
TOTAL	76.4 ± 27.9 ^a^	56.0 ± 25.4 ^b^	55.8 ± 32.3 ^bc^	32.3 ± 24.1 ^c^

Results were adjusted by energy intake, gender, and BMI. Groups showing different superscript letters differ significantly (*p* < 0.05).

**Table 3:** Mean daily intake of the major food categories and nutritional compounds related with immune function, according to age.

	Age Group (Years)			
	<50	50–65	66–80	>80
Energy (kcal/day) ^#^ Food groups (g)	1922 ± 575 ^a,b^	2033 ± 581 ^a^	1624 ± 435 ^b^	1728 ± 400 ^a,b^
Cereals	176.3 ± 9.7 ^a^	185.0 ± 9.0 ^a^	143.9 ± 15.8 ^a^	153.5 ± 13.4 ^a^
Dairy products	311.7 ± 29.8 ^a^	396.7 ± 27.7 ^a^	410.8 ± 48.6 ^a^	408.0 ± 41.3 ^a^
Fats and oils	25.2 ± 2.4 ^a^	30.1 ± 2.2 ^a^	23.8 ± 3.9 ^a^	24.0 ± 3.3 ^a^
Sugar/sugary products **	29.7 ± 2.8 ^a^	11.0 ± 2.6 ^b^	13.8 ± 4.6 ^b^	11.2 ± 3.9 ^b^
Vegetables *	157.6 ± 19.0 ^a,b^	205.2 ± 17.6^a^	164.0 ± 31.0 ^a,b^	105.1 ± 26.2 ^b^
Potatoes **	45.8 ± 6.8 ^a,b^	43.1 ± 6.3 ^a^	69.8 ± 11.3 ^b^	68.6 ± 9.4 ^a,b^
Legumes	28.7 ± 12.4 ^a^	37.8 ± 11.5 ^a^	27.5 ± 20.1 ^a^	21.5 ± 17.1 ^a^
Fruit **	193.8 ± 27.6 ^a^	343.9 ± 25.6 ^b^	271.4 ± 45.0 ^a,b^	205.2 ± 38.2 ^a^
Meat and derivate	134.8 ± 7.9 ^a^	115.5 ± 7.3 ^a^	110.9 ± 12.9 ^a^	122.9 ± 10.9 ^a^
Fish and derivate **	42.2 ± 5.5 ^a^	60.7 ± 5.1 ^a,b^	88.2 ± 8.9 ^b^	89.1 ± 7.6 ^b^
Non-alcoholic drinks **	307.3 ± 27.6 ^a^	167.3 ± 25.7 ^b^	182.8 ± 45.0 ^b^	145.0 ± 38.2 ^b^
Dietary components				
Carbohydrates (g)	206.9 ± 4.9 ^a^	199.2 ± 4.6 ^a^	196.8 ± 8.1 ^a^	188.9 ± 6.8 ^a^
Total fiber **	17.5 ± 0.7 ^a^	22.1 ± 0.7 ^b^	19.3 ± 1.2 ^a^	17.5 ± 1.0 ^a^
Insoluble fiber **	11.6 ± 5.6 ^a^	14.3 ± 5.4 ^b^	12.4 ± 4.0 ^a,b^	10.2 ± 3.8 ^a^
Soluble fiber **	2.1 ± 0.8 ^a^	2.9 ± 1.3 ^b^	2.6 ± 0.8 ^a,b^	2.1 ± 0.9 ^a^
Proteins (g)	83.8 ± 2.5 ^a^	89.6 ± 2.3 ^a^	89.7 ± 4.0 ^a^	90.9 ± 3.4 ^a^
Animal	55.3 ± 2.5 ^a^	58.6 ± 2.3 ^a^	59.9 ± 4.0 ^a^	61.1 ± 3.4 ^a^
Vegetal	25.0 ± 1.1 ^a^	29.2 ± 1.0 ^a^	27.6 ± 1.8 ^a^	27.9 ± 1.6 ^a^
Lipids (g)	78.3 ± 2.1 ^a^	78.3 ± 2.0 ^a^	79.2 ± 3.5 ^a^	85.4 ± 3.0 ^a^
SFAs **	24.8 ± 1.0 ^a^	25.1 ± 0.9 ^a^	28.0 ± 1.6 ^a,b^	30.4 ± 1.4 ^b^
MUFAs **	31.0 ± 1.4 ^a,b^	34.9 ± 1.3 ^a^	28.9 ± 2.3 ^b^	28.5 ± 2.0 ^b^
PUFAs **	16.1 ± 1.0 ^a,b^	11.9 ± 0.9 ^a^	15.7 ± 1.7 ^a,b^	19.6 ± 1.4 ^b^
Vitamins				
Carotenes (μg) **	2048 ± 1583 ^a^	2464 ± 1821 ^a^	1899 ± 2278 ^a,b^	1014 ± 315 ^b^
Folic acid (μg) **	308.3± 128.1 ^a^	390.8 ± 177.6 ^b^	330.3 ± 128.4 ^a^	275.3 ± 82.64 ^a^
Vitamin A (μg) **	822.2 ± 477.8 ^a^	743.2 ± 435.5 ^a^	619.7 ± 425.3 ^b^	430.7 ± 129.8 ^b^
Vitamin B_12_ (μg)	7.7 ± 7.0 ^a^	7.1 ± 4.3 ^a^	8.2 ± 3.1 ^a^	8.7 ± 2.6 ^a^
Vitamin B_6_ (mg) **	1.9 ± 0.7 ^a^	2.2 ± 0.7 ^b^	2.1 ± 0.6 ^a,b^	1.8 ± 0.4 ^a^
Vitamin C (mg) **	123.7 ± 72.2 ^a^	199.6 ± 165.2 ^b^	187.2 ± 97.2 ^a,b^	155.2 ± 74.8 ^a^
Vitamin D (μg) **	2.8 ± 1.9 ^a^	3.8 ± 2.6 ^b^	1.7 ± 1.7 ^a,c^	0.8 ± 0.3 ^c^
Vitamin E (mg) **	12.7 ± 6.8 ^a^	10.5 ± 5.0 ^a^	12.1 ± 5.1 ^a^	13.7 ± 5.5 ^a^
Minerals				
Copper (mg)	1.4 ± 0.7 ^a^	1.3 ± 0.5 ^a^	1.3 ± 0.4 ^a^	1.1± 0.3 ^a^
Iron (mg)	12.2 ± 5.2 ^a^	12.8 ± 3.9 ^a^	13.2 ± 3.7 ^a^	12.6 ± 2.7 ^a^
Selenium (μg) *	118.9 ± 52.8 ^a^	122.0 ± 42.4 ^a^	136.5 ± 38.3 ^a^	137.6 ± 33.9 ^a^
Total polyphenols (mg) **	1202 ± 651 ^a^	1721 ± 938 ^b^	1265 ± 1324 ^a^	841 ± 658 ^a^

Results derived from multivariate analysis adjusted by energy intake, gender and BMI (Asterisks denotes variables showing statistically significant effects (* *p* < 0.05; ** *p* < 0.01) on the multivariate model). ^#^ Unadjusted univariate model. Variables are presented as estimated marginal mean ± sd. SFAs, saturated fatty acids. MUFAs, monounsaturated fatty acids. PUFAs, polyunsaturated fatty acids. Groups showing different superscript letters differ significantly (*p* < 0.05; Bonferroni’s test).

## Supplementary Materials

The following are available online at https://www.mdpi.com/2072-6643/11/8/1765/s1, Table S1: Exclusion criteria used in the present study.
