# Supplementary material for: Age-Associated Changes in Gut Microbiota and Dietary Components Related with the Immune System in Adulthood and Old Age: A Cross-Sectional Study
Source: Nutrients. 2019 Jul 31;11(8):1765. doi: 10.3390/nu11081765 (PMC6722604; doi:10.3390/nu11081765)
Supplement: Supplementary file 1 [file nutrients-11-01765-s001.pdf]

Supplementary Table 1. Exclusion criteria used in the present study.

**Exclusion criteria**	
Previous diagnosis of:	• Gastrointestinal cancer<br>• Inflammatory bowel disease<br>• Allergy, diabetes type II<br>• Other autoimmune disease<br>• Morbid obesity<br>• Any disease requiring gastrointestinal surgery
Consumption during the previous month of:	• Food supplements<br>• Probiotic or prebiotic supplemented foods (standard yogurt consumption was allowed)<br>• Antibiotics<br>• Oral glucocorticoids<br>• Immunotherapy
